# Evaluation of cooling effect and road performance of warm mixed high viscosity porous asphalt mixture

**DOI:** 10.1371/journal.pone.0307554

**Published:** 2024-07-29

**Authors:** Jun’an Lei, Nanxiang Zheng, Zhiyuan Jia

**Affiliations:** 1 School of Civil Engineering and Architecture, HuBei University of Arts and Science, Xiangyang, Hubei, China; 2 Hubei Superior and Distinctive Discipline Group of “New Energy Vehicle and Smart Transportation”, Xiangyang, Hubei, China; 3 School of Highway, Chang’an University, Xi’an, Shaanxi, China; Shandong University of Technology, CHINA

## Abstract

In order to study the effect of different warm mixing agents on the cooling and road performance of porous mixture bonded with High Viscosity Asphalt (HVA), the Superpave Gyratory Compactor (SGC) was proposed to conduct variable temperature compaction test on the warm mixed porous asphalt mixture. The relationship between the compaction difficulty coefficient and the temperature of the mixture was obtained by regression, and the mixing and compacting temperature and cooling effect of the warm mixed porous asphalt mixture were determined. Then, the influence of Evotherm M1 and EC120 warm mix agents on the performance of porous asphalt mixture was compared by rutting tests, beam bending tests, immersion Marshall tests, freeze-thaw splitting tests, and penetration strength tests. The results show that two types of warm mixing agents can reduce the mixing and rolling temperatures of the mixture by more than 10°C. The addition of warm mix agents will increase the high temperature stability, water stability, and shear resistance of porous asphalt mixtures. For the improvement effect of high temperature stability, EC120 warm mixing agent is greater than Evotherm M1 warm mixing agent, and for the improvement effect of water stability, Evotherm M1 warm mixing agent is greater than EC120 warm mixing agent. For shear resistance, the improvement effect of both is equivalent, with a penetration strength increase of about 3% to 4%. For low temperature performance, Evotherm M1 warm mixing agent improved the low temperature performance, while EC120 warm mixing agent showed the opposite effect.

## 1 Introduction

Porous asphalt pavement generally refers to the pavement with a void ratio of more than 15%. Compared with ordinary dense-graded asphalt pavement, it can quickly remove water on the road surface in rainy days to ensure the safety of driving [1]. In addition, due to its large connecting pores, the noise generated by the vehicle during driving is relatively small [2], which has good driving comfort. However, due to the lack of filling effect of fine aggregate, the porous asphalt mixture is easy to appear water damage such as looseness and potholes. In order to improve road performance, high viscosity modified asphalt is usually used to increase the bonding degree between aggregates, thereby reducing the occurrence of water damage [3, 4]. Due to the high viscosity of the modified asphalt, the construction temperature also increases accordingly, which not only increases the consumption of energy, but also easily leads to asphalt aging. Therefore, the use of warm mixing technology is one of the important ways to reduce energy consumption and emissions, and improve construction workability [5, 6]. At present, the commonly used warm mixing agents mainly include organic viscosity reducer, chemical additives and asphalt foam type [7, 8], which can effectively reduce the mixing and compaction temperature of asphalt mixture.

Many scholars have carried out studies on the influence of warm mixing agent on the pavement performance of HVA and porous asphalt mixture [9–11], but the evaluation method of the cooling effect of warm mixing agent on HVA is still insufficient. The commonly used methods to evaluate the cooling performance of warm mix agent mainly include the viscosity-temperature curve method of asphalt [12, 13] and the variable temperature compaction method of asphalt mixture [14, 15]. Among them, the viscosity-temperature curve method is reasonable to evaluate the matrix asphalt. Some studies have shown that for polymer modified asphalt, the mixing and compaction temperature of the mixture determined by the viscosity-temperature curve method is usually higher than 195°C [16], and the warm mixing agent cannot achieve the effect of cooling. Therefore, it is inappropriate to use this method to determine the construction temperature of the high viscosity modified asphalt mixture. The variable temperature compaction method is to conduct compaction tests on asphalt mixtures by changing the temperature, and then draw the relationship curve between the porosity of asphalt mixtures and temperature to determine the cooling performance. However, the design method of large void asphalt mixture is not Marshall compaction test, and the compaction process is not consistent with the kneading process of rollers in road construction. Therefore, this method also has shortcomings in evaluating the cooling effect of porous asphalt mixture.

In order to study the effect of warm mixing agents on the cooling effect and road performance of porous asphalt mixtures, the variable temperature rotary compaction method was proposed to evaluate the cooling effect based on the variable temperature compaction method. On the basis of the compaction difficulty coefficient proposed by Yan Xili [17], a regression model of the relationship between compaction difficulty coefficient and temperature was obtained to determine the cooling effect of warm mix high viscosity modified porous asphalt mixture. On this basis, the influence of warm mixing agents on the road performance of high viscosity modified porous asphalt mixture was studied through rutting tests, beam bending tests, immersion Marshall tests, freeze-thaw splitting tests, and penetration strength tests. The research results have good reference value for improving the application of warm mix porous asphalt mixtures.

## 2 Raw materials and asphalt mixture preparation

### 2.1 Raw materials

#### 2.1.1 Asphalt

The asphalt used is HVA, and the basic technical indicators are shown in Table 1.

**Table 1:** Technical properties of HVA.

Index	Unit	Requirement	Test result	Test method
Penetration (25°C, 100g, 5s)	0.1mm	≥40	48	T0604
Softening point	°C	≥80	100.45	T0606
Solubility	%	≥99	99.3	T0607
Brookfield viscosity (135°C)	Pa•s	--	4.125	T0625
60°C dynamic viscosity	Pa•s	≥50000	896100	T0620
Elastic recovery (25°C)	%	≥95	99.78	T0662

#### 2.1.2 Warm mixing agent

Organic viscosity reducing agent EC120 made in China and surfactant Evotherm M1 made in the United States were selected as the warm mixing agent. Their properties are shown in Table 2.

**Table 2:** Types of warm mixing agents.

Type	Producer	Physical state	Color	Working mechanism
EC120	China	Solid powder	White	Organic viscosity reducers
Evotherm M1	United States	Liquid	Dark brown	Surfactants

#### 2.1.3 Aggregates

The lithology of coarse and fine aggregates is diabase, and the mineral powder is limestone. The coarse aggregates are divided into three specifications: 10-15mm, 5-10mm, and 3-5mm, while the fine aggregates are 0-3mm machine-made sand. The technical indicators of coarse and fine aggregates are shown in Tables 3 and 4.

**Table 3:** Technical indicators of coarse aggregate.

Index	Unit	Requirement	Test result	Test method
Apparent relative density	--	≥2.60	2.98	T0328
Crushing value	%	≤24	6.89	T0316
Los Angeles wear loss	%	≤28	8.23	T0317
Polished stone value	--	≥42	52	T0321
Soundness	%	≤8	3.8	T0314
Elongated particle contents	%	≤10	8.2	T0312

**Table 4:** Technical indicators of fine aggregate.

Index	Unit	Requirement	Test result	Test method
Apparent relative density	--	≥2.50	2.956	T0328
Soundness (>0.3mm)	%	≤10	5.6	T0340
Mud content (<0.075mm)	%	≤1	0.3	T0333
Sand equivalent	%	≥65	85	T0334
Angularity	s	≥30	45.7	T0345

### 2.2 Preparation of porous asphalt mixture

**Fig 1:**
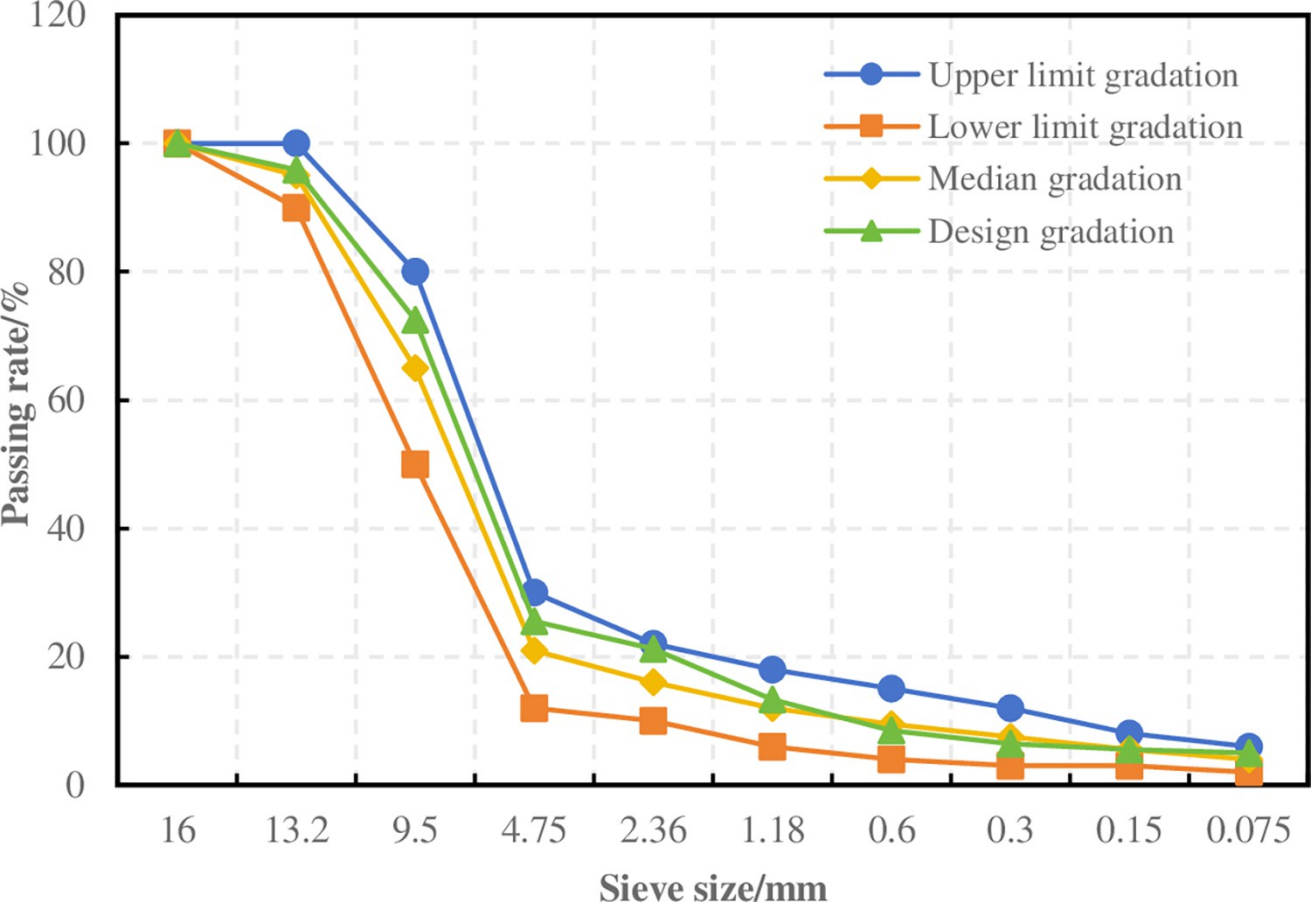
Grading curve of aggregates.

The gradation selected for the preparation of porous asphalt mixture is shown in Fig 1.

**Fig 2:**
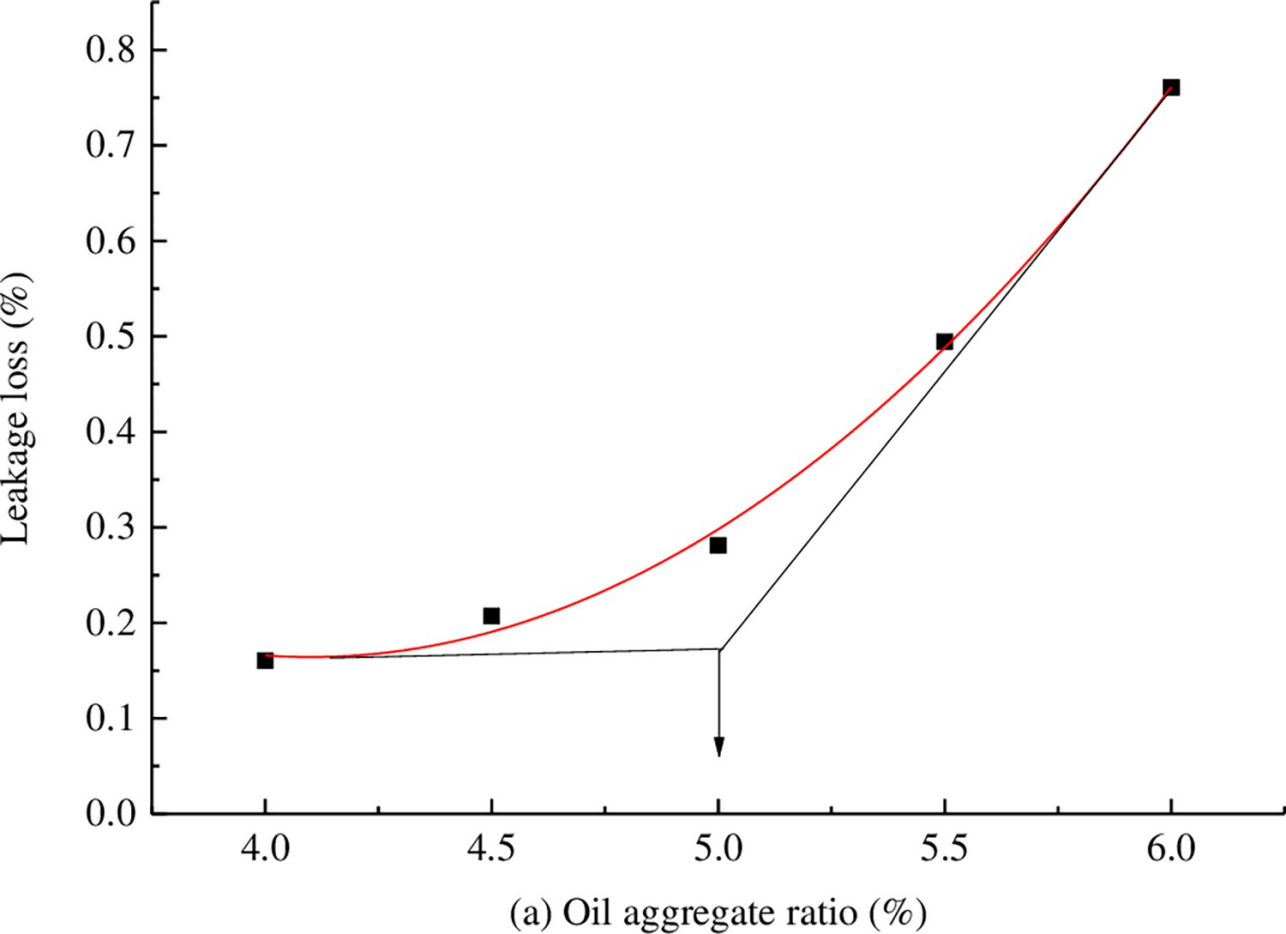
Leakage test results.

**Fig 3:**
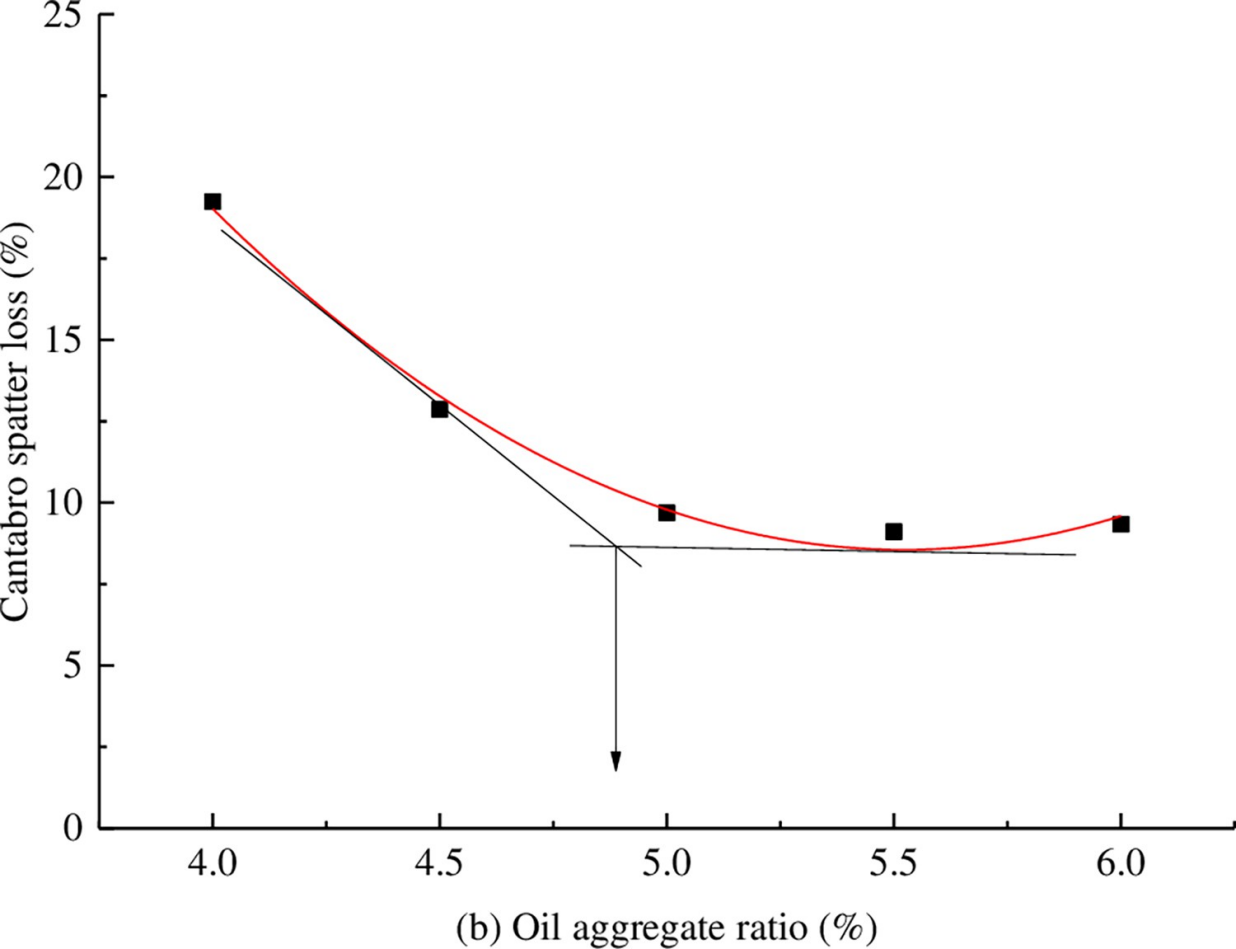
Kentucky scattering test results.

Marshall specimens were formed with oil-stone ratios of 4%, 4.5%, 5%, 5.5%, and 6%, and leakage tests and Kentucky scattering tests were conducted to comprehensively determine the asphalt dosage. The results of the leakage test and Kentucky scattering test are shown in Figs 2 and 3.

From Figs 2 and 3, it can be seen that the optimal range of oil-stone ratio is 4.9% to 5.0%. In order to ensure good durability of the large void asphalt mixture, the thickness of the asphalt film should be increased. Therefore, 5.0% is chosen as the optimal oil-stone ratio.

## 3 Experimental

In order to study the effect of warm mix agents on the cooling effect and road performance of porous asphalt mixtures, three sets of schemes were designed as shown in Table 5. According to the manufacturer’s recommendation and existing research, the dosage of EC120 warm mix agent is 4% of the asphalt mass, and the dosage of Evotherm M1 warm mix agent is 0.6% of the asphalt mass. On this basis, comparative experimental studies were conducted on the cooling performance, high temperature performance, low temperature performance, water stability performance, and shear strength of warm mix asphalt mixture.

**Table 5:** Comparative test scheme.

Type of mixture	Binder	Optimal oil-stone ratio
OGFC-13①	HVA	5.0%
OGFC-13②	HVA+0.6% Evotherm M1	5.0%
OGFC-13③	HVA+4% EC120	5.0%

### 3.1 Evaluation of cooling performance

The exponential relation between the compaction degree K of asphalt mixture and the compaction number N was obtained by Yan Xili [17] through regression.

$$K=ae^{-\frac{b}{N+1}}$$ (1)

Where, a and b are regression parameters.

$$K_{\infty }=\underset{N\to \infty }{\lim}(K)=a$$ (2)

$$K_{0}=\underset{N\to 0}{\lim}(K)=K_{\infty }e^{-b}$$ (3)

**Fig 4:**
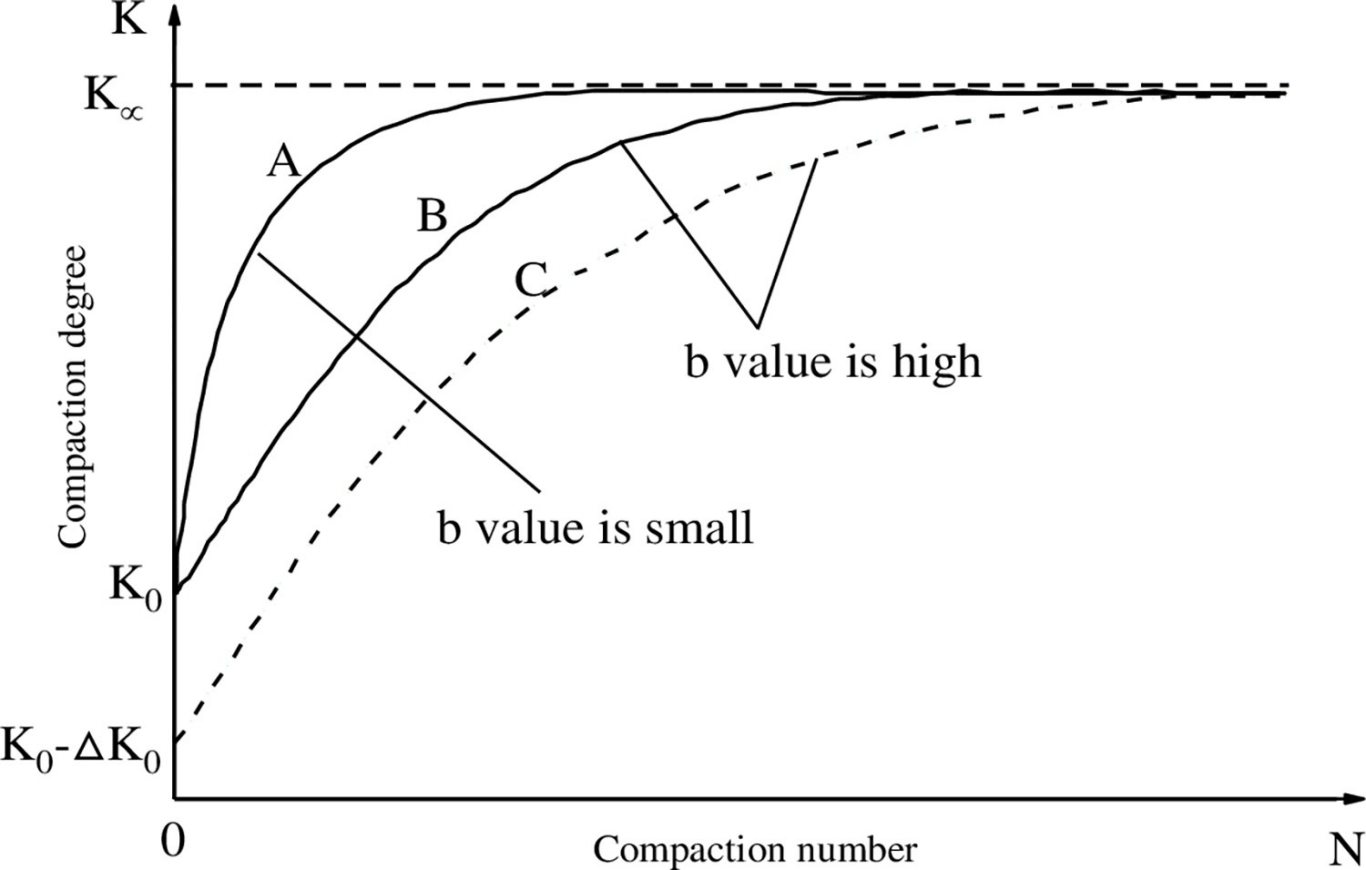
Limit conditions and geometric properties of the mixture compaction curves.

Where, K_∞_ is the ultimate maximum compaction degree of asphalt mixture; K_0_ is the compaction degree of asphalt mixture under natural packing state. The relationship curve between K and N determined by Eq (1) is shown in Fig 4.

Curves A and B have the same K_0_ or K_∞_-K_0_, but the curvature factor b is different, resulting in a different growth process of compactness. Curves B and C have the same curvature factor b, but K_0_ or K_∞_-K_0_ are different, which also leads to a different growth process of compactness. Therefore, K_∞_-K_0_ and b simultaneously determine the variation characteristics of the K-N curve. According to this, the compacting difficulty factor μ of asphalt mixture is defined.


$$\mu =(K_{\infty }-K_{0})b$$ (4)

The larger the μ value, the more difficult the asphalt mixture is to be compacted, and the smaller the μ value, the easier the asphalt mixture is to be compacted.

In this study, the μ value of warm mixing asphalt mixture was obtained by SGC rotary compaction method, and the cooling effect of warm mixing agent on porous asphalt mixture was evaluated. The SGC specimen is formed in a height control mode. The specimen is a cylinder with a height of 100mm and a diameter of 100mm. The vertical pressure is 0.6MPa, the tilt angle is 1.16°, and the rotary compaction speed is 30r/min. When the height of the specimen reaches 100mm, the compaction will stop automatically. The compaction temperatures of SGC were 150°C, 160°C, 170°C, 180°C and 190°C, respectively. The asphalt binder was HVA asphalt, warm mixed HVA asphalt mixed with 0.6% Evotherm M1 and 4% EC120. In addition, a control group was added as SBS modified asphalt. The instrument will automatically record multiple sets of data during the testing process, including compaction times, specimen height, and real-time density of specimens. The compaction degree K_N_ of the mixture during the compaction process is defined as the percentage of the density of the specimen during the compaction process to the theoretical maximum density of the mixture, as shown in Eq (5).

$$K_{N}=\frac{\rho_{N}}{\rho_{\mathrm{ti}}}\times 100\%$$ (5)

Where, K_N_-the compaction degree of the mixture when the number of compaction cycles is N, %; ρ_N_-The density of the mixture when the number of compaction cycles is N, g/cm^3^; ρ_Ti_—The maximum theoretical density of the mixture, g/cm^3^.

**Fig 5:**
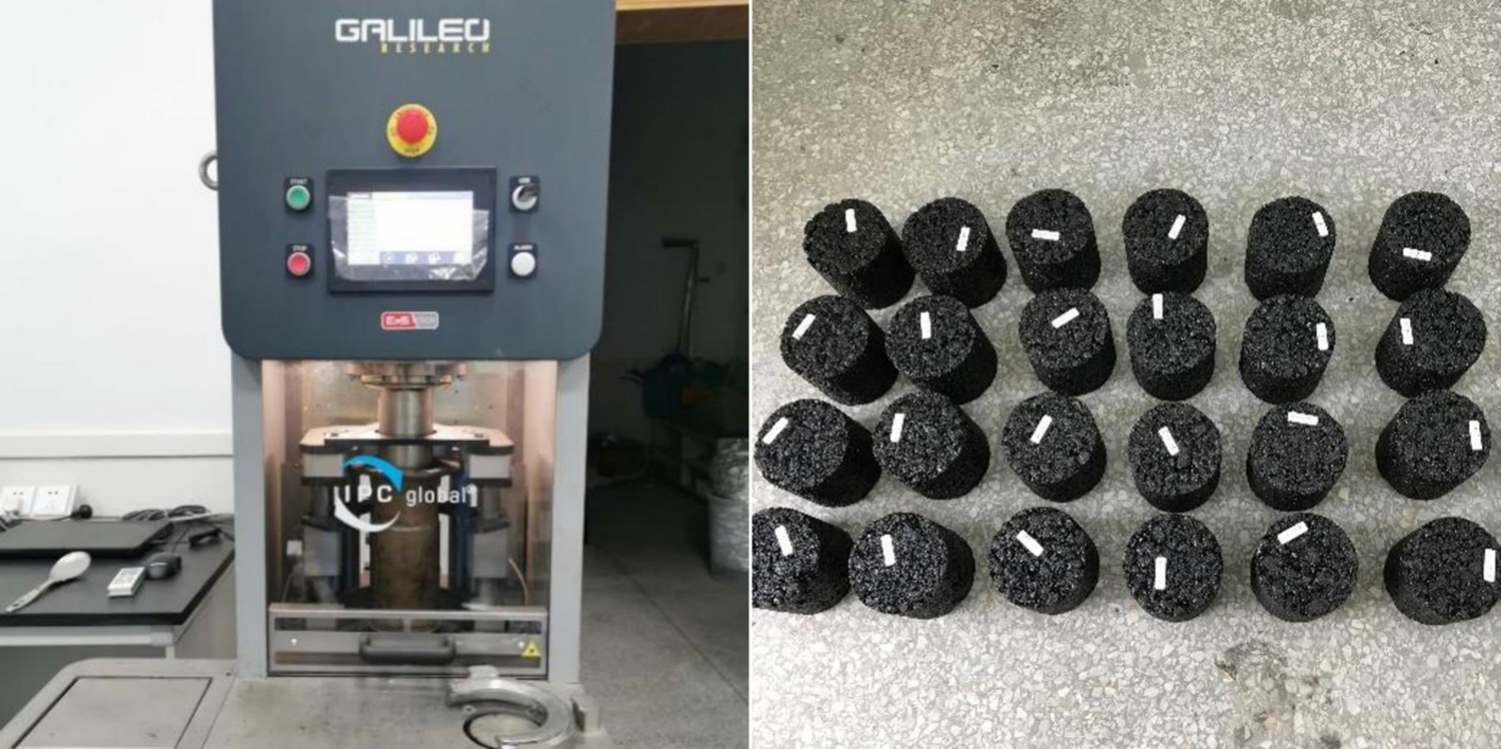
SGC rotary compaction test.

The K-N curve was obtained by SGC compaction test, and then the compacting difficulty coefficient μ was calculated by Eqs 1–4. The SGC rotary compaction test is shown in Fig 5.

### 3.2 High temperature performance

**Fig 6:**
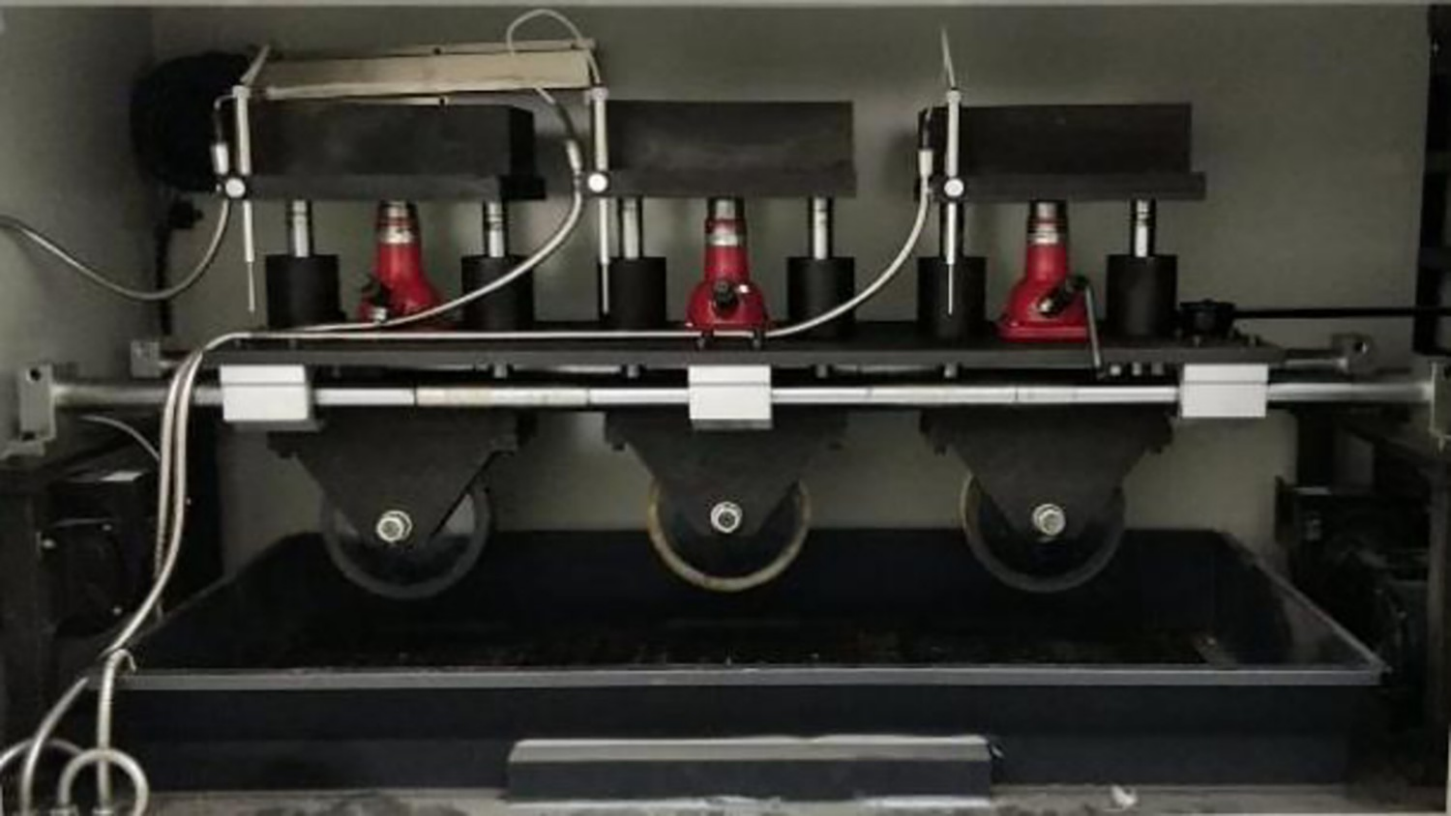
Rutting test of asphalt mixture.


The rutting test is used to evaluate the high temperature stability of warm mixed porous asphalt mixture. The test was carried out according to Section T0719 of specification JTG E20-2011 [18]. Firstly, according to the plan, three sets of rut plate specimens with dimensions of 300mm × 300mm × 50mm will be formed. Then, the test pieces were placed in the rutting machine and kept at 60°C for 5h. Finally, the instrument was started to roll the specimen repeatedly and record the deformation automatically, and the wheel pressure was set to 0.7MPa±0.05MPa. The rutting test is shown in Fig 6. The dynamic stability DS of rutting test results is calculated according to the Eq (6).

$$DS=\frac{(t_{2}-t_{1})\times N}{d_{2}-d_{1}}\times C_{1}\times C_{2}$$ (6)

Where: DS—Dynamic stability of asphalt mixture, times /mm; d_1_—The deformation corresponding to t_1_ time, mm; d_2_—The deformation corresponding to t_2_, mm; C_1_—Type coefficient of testing machine, 1.0; C2—Specimen coefficient, 1.0; N—Rolling speed of the test wheel, 42 times /min.

### 3.3 Low temperature performance

The low temperature performance of the mixture was studied by the low temperature bending test of the beam. The test was carried out according to Section T0715 of the JTG E20-2011 specification [18]. The UTM-130 hydraulic servo asphalt material test system was used for testing, and the test temperature was controlled at -10°C. The beam specimen was cut from the rutting plate, with dimensions of (250mm ± 2mm) x (30mm ± 2mm) x (35mm ± 2mm) and a span of 200mm ± 0.5mm. The loading rate during the test was 50mm/min. The flexural tensile strength, maximum flexural tensile strain, and flexural stiffness modulus were used to evaluate the low temperature performance of asphalt mixtures, and each index was calculated according to Eqs (7)–(9).

$$R_{B}=\frac{3LP_{B}}{2bh^{2}}$$ (7)

$$\varepsilon_{B}=\frac{6hd}{L^{2}}$$ (8)

$$S_{B}=\frac{R_{B}}{\varepsilon_{B}}$$ (9)

Where: R_B_- Flexural tensile strength, MPa; ε_B_- Maximum flexural tensile strain; S_B_- Modulus of bending stiffness, MPa; b- Width of the specimen span, mm; h- Height of the specimen, mm; L- Span of specimen, mm; P_B_- The maximum load when the specimen is damaged, N; d- Mid-span deflection at the time of specimen failure, mm.

### 3.4 Water stability

The water stability of the mixture was studied by immersion Marshall test and freeze-thaw splitting test.

#### 3.4.1 Immersion Marshall test

According to Section T0709 of JTG E20-2011 specification [18], a cylindrical Marshall standard specimen with a diameter of 101.6mm and a height of 63.5mm was formed before the test. Firstly, the asphalt mixture specimen was immersed in a water bath at 60°C for 48h, and then the Marshall stability of the specimen before and after immersion was tested by an automatic Marshall tester. Finally, the residual stability was calculated according to Eq (10).

$$MS_{0}=\frac{MS_{1}}{MS}\times 100$$ (10)

Where: MS_0_- Residual stability of the specimen, %; MS_1_- Stability of the specimen after 48h immersion in water, kN; MS- Stability of the specimen, kN.

#### 3.4.2 Freeze thaw splitting test

According to Section T0729 of JTG E20-2011 specification [18], a cylindrical Marshall standard specimen with a diameter of 101.6mm and a height of 63.5mm was formed before the test. The splitting strength of the Marshall specimen before and after the freeze-thaw cycle was tested respectively, and the ratio of freeze-thaw splitting strength was calculated according to Eq (11).

$$TSR=\frac{{\bar{R}}_{T2}}{{\bar{R}}_{T1}}\times 100$$ (11)

Where: TSR—ratio of freeze-thaw splitting strength, %; ${\bar{R}}_{T2}$-Average splitting strength of the specimen after freeze-thaw cycle, MPa; ${\bar{R}}_{T1}$-Average splitting strength of specimens without freeze-thaw cycle, MPa.

### 3.5 Uniaxial penetration strength test

**Fig 7:**
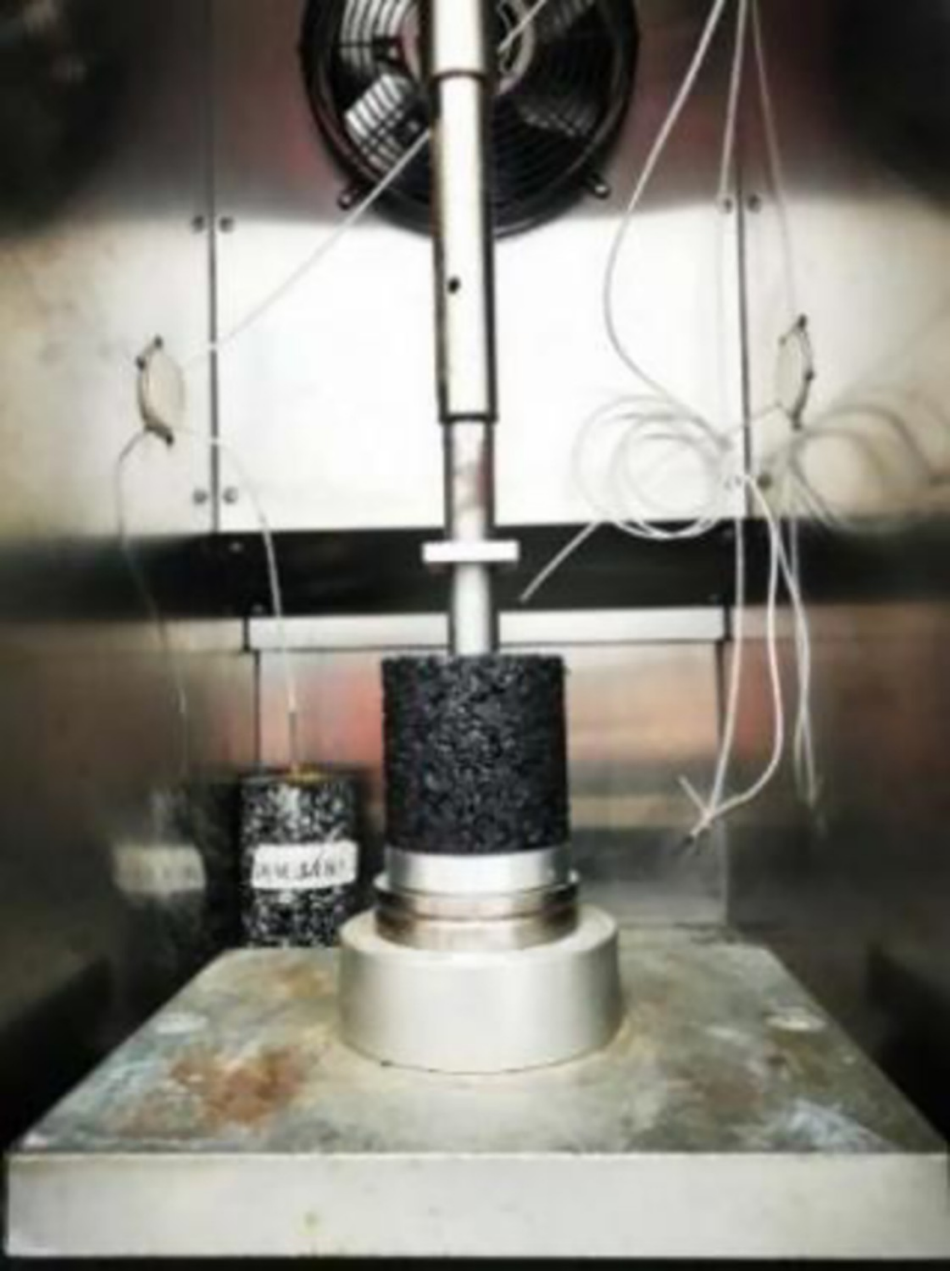
Uniaxial penetration test.

The penetration strength measured by uniaxial penetration test can reflect the shear resistance of asphalt mixture. The mixture specimen used in the test was formed by SGC rotary compactor, with a height of 100mm and a diameter of 100mm. The test was carried out by UTM-130 hydraulic servo asphalt material test system, as shown in Fig 7.

The test temperature was 60°C, the loading rate was 1mm/min, and the test was stopped when the stress value dropped to 90% of the extreme stress point. The penetration strength of the mixture was calculated according to Eq (12) and (13).

$$R_{\tau }=f_{\tau }\sigma_{P}$$ (12)

$$\sigma_{P}=\frac{P}{A}$$ (13)

Where: R_τ_- Penetration strength, MPa; σ_p_- Penetration stress, MPa; P- The ultimate load when the specimen is damaged, N; A- The cross section area of the head, 637.9mm^2^; f_τ_- The penetration stress coefficient, 0.34.

## 4 Results and discussion

### 4.1 Cooling performance

**Fig 8:**
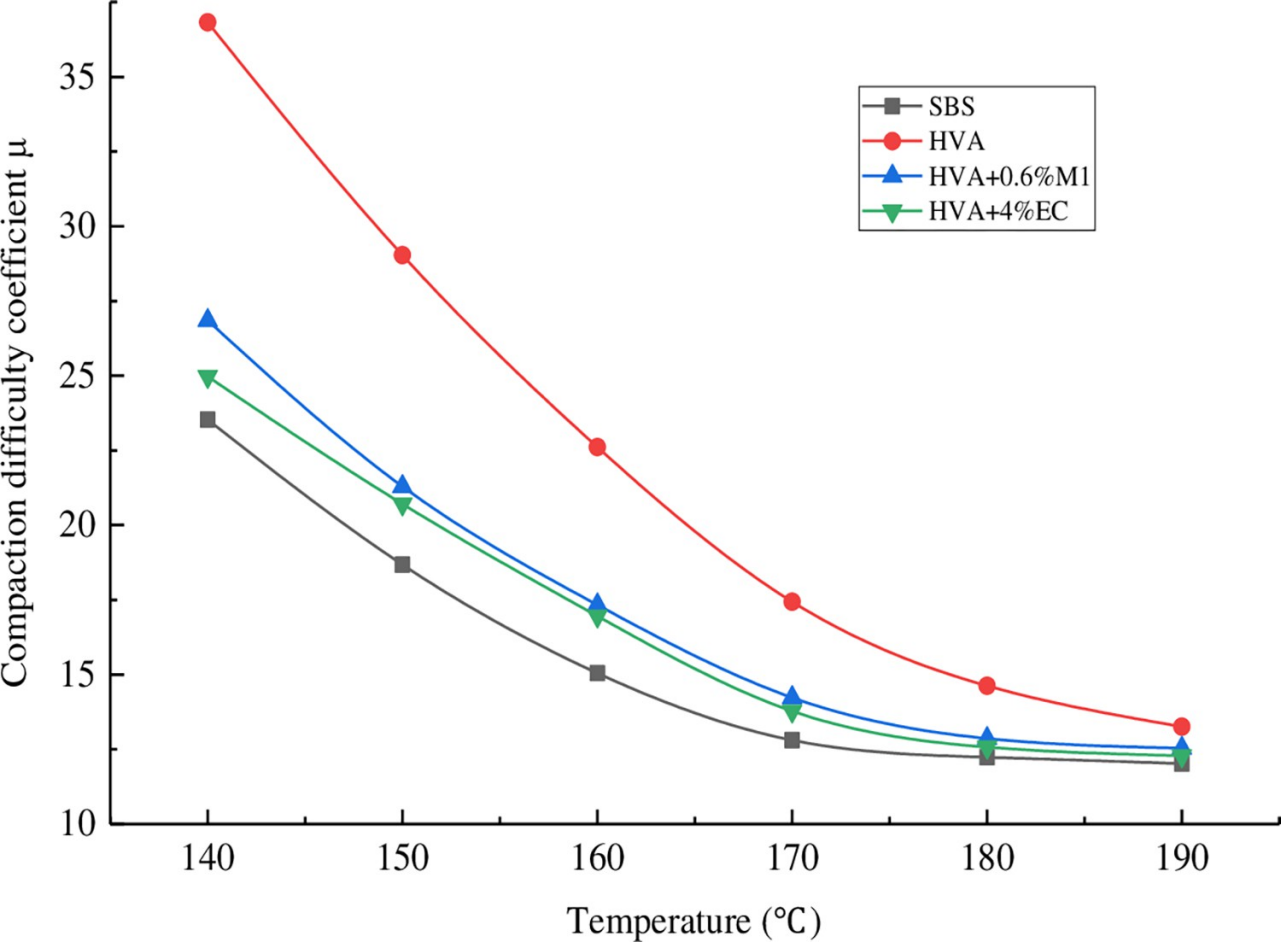
Relationship between compaction difficulty coefficient and temperature.

The relationship between the rotary compaction difficulty coefficient μ and temperature of asphalt mixture is shown in Fig 8.

**Fig 9:**
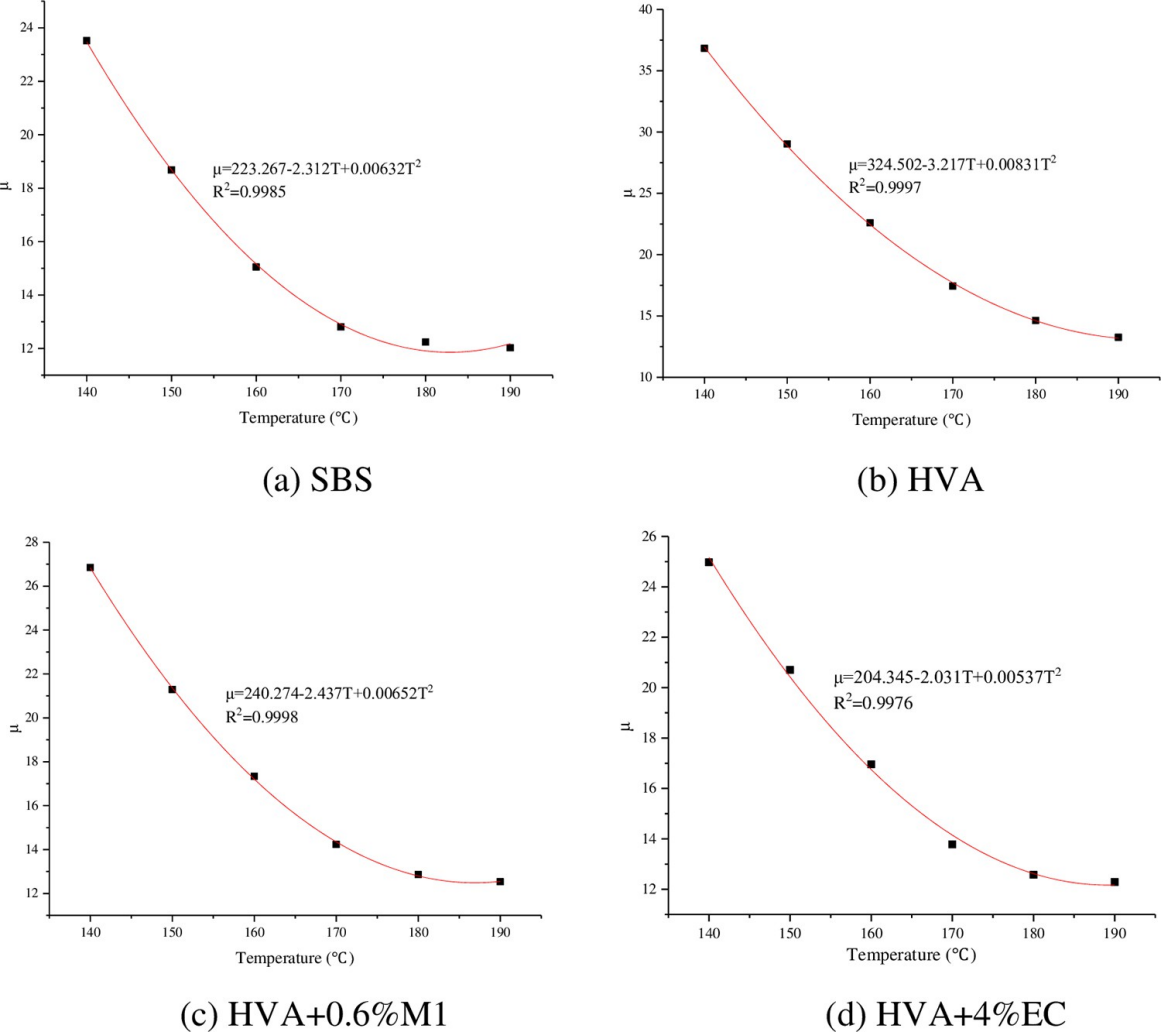
Fitting results of compacting difficulty coefficient and temperature.

It can be seen from Fig 8 that the compacting difficulty coefficients of all asphalt mixtures decrease with the increase of temperature, because the viscosity of asphalt decreases with the increase of temperature, so the mixture is more likely to be compacted. At the same temperature, the coefficient of compaction difficulty of HVA mixture is the largest. When the warm mixing agent was added, the coefficient of compaction difficulty was reduced. It can be seen that the warm mixing agent contributes to the compaction of the mixture, mainly because the warm mixing agent plays a lubricating role. SBS modified asphalt mixture has the lowest compactibility coefficient because of its lowest viscosity. The relationship between the compaction difficulty coefficient and temperature was obtained through fitting, as shown in Fig 9 and Table 6.

**Table 6:** Regression equation between compaction difficulty coefficient and temperature.

Types of asphalt	Regression equation
SBS	μ = 223.267–2.312T+0.00632T^2^
HVA	μ = 324.502–3.217T+0.00831T^2^
HVA+0.6% M1	μ = 240.274–2.437T+0.00652T^2^
HVA+4%EC	μ = 204.345–2.031T+0.00537T^2^

In actual engineering, the mixing temperature of SBS modified asphalt mixture shall not be lower than 165°C and the compaction temperature shall not be lower than 150°C. Therefore, 155°C and 165°C are used as the mixing and compaction standards for modified asphalt to determine the construction temperature of HVA mixture.

By substituting T = 155°C and T = 165°C into the regression equations of SBS modified asphalt mixture, the compaction difficulty coefficients of SBS modified asphalt mixture at compaction and mixing temperatures were obtained to be 15.191 and 13.223, respectively. Then, μ = 15.191 and μ = 13.223 were respectively inputted into the regression equations of HVA, HVA+0.6% M1, and HVA+4% EC to obtain the compaction and mixing temperatures of each asphalt mixture. The calculation results are shown in Table 7.

**Table 7:** Construction temperature of asphalt mixture.

Construction temperature	SBS	HVA	HVA+0.6% M1	HVA+4%EC
Mixing temperature (°C)	165.0	184.4	172.8	172.2
Compaction temperature (°C)	155.0	175.9	164.8	163.8

From Table 7, it can be seen that the mixing and compaction temperature of HVA asphalt mixture is the highest, about 20°C higher than that of SBS modified asphalt mixture. After adding 0.6% Evotherm M1 warm mix agent, the mixing and compaction temperature of asphalt mixture decreased by 11.6°C and 11.1°C compared to HVA asphalt mixture. After adding 4% EC120 warm mix agent, the mixing and compaction temperature of asphalt mixture decreased by 12.2°C and 12.1°C compared to HVA asphalt mixture. Therefore, both EC120 and Evotherm M1 warm mix agents can reduce the construction temperature of HVA mixtures.

### 4.2 Rutting test

**Fig 10:**
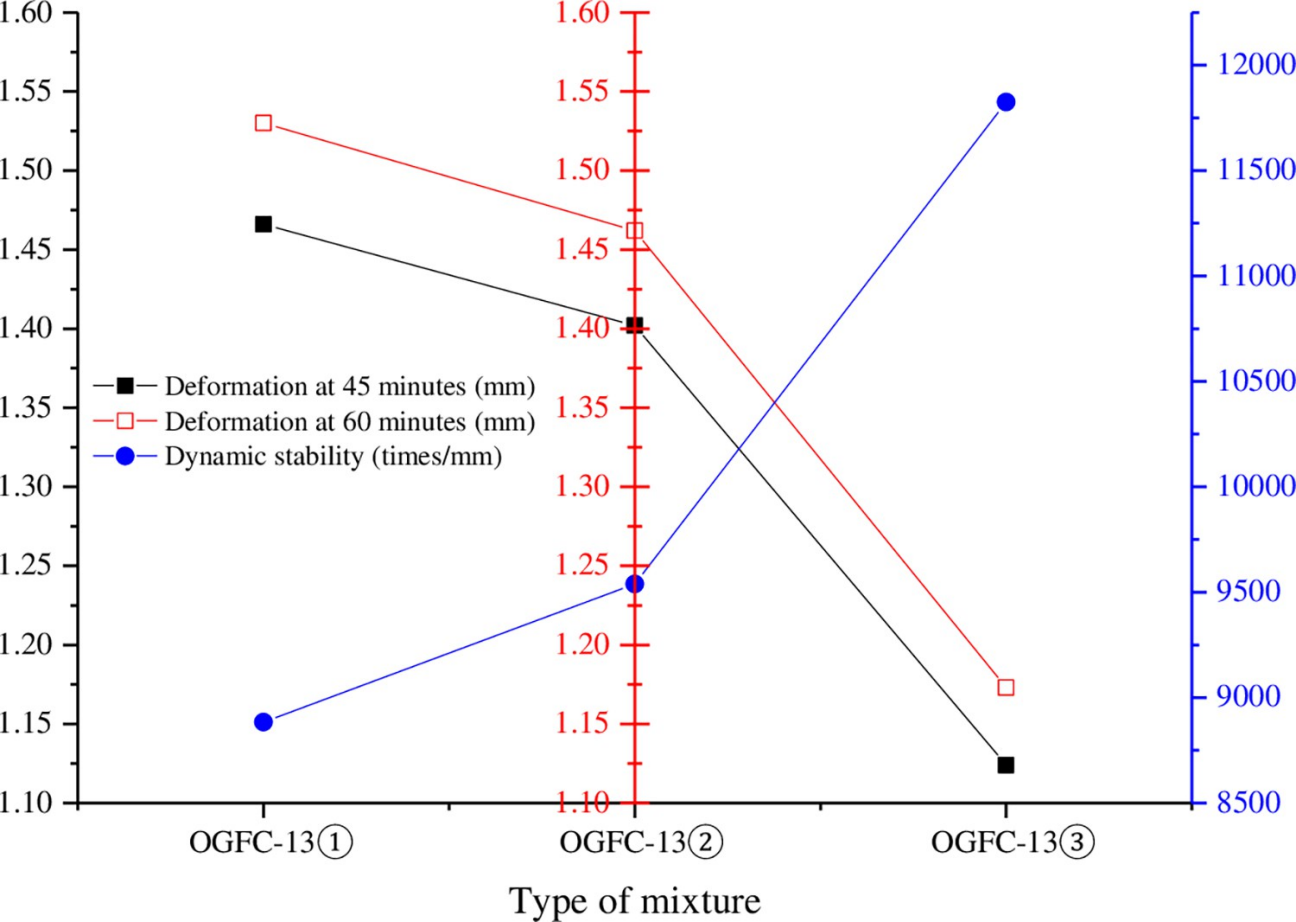
Comparison of rutting test results.

The rutting test results of each mixture are shown in Table 8 and Fig 10.

**Table 8:** Results of rutting test.

Type of mixture	Deformation at 45 minutes (mm)	Deformation at 60 minutes (mm)	Dynamic stability (times/mm)
OGFC-13①	1.466	1.530	8884
OGFC-13②	1.402	1.462	9539
OGFC-13③	1.124	1.173	11825

It can be seen from Table 8 and Fig 10 that the rutting deformation of the mixture at 45min and 60min is OGFC-13①> OGFC-13②> OGFC-13③, indicating that the deformation resistance of the warm mixed asphalt mixture is enhanced. The dynamic stability is far greater than the requirement of not less than 3000 for heavy traffic sections, and OGFC-13①< OGFC-13②< OGFC-13③, indicating that the warm mixing agent will increase the high temperature stability of the mixture. The dynamic stability of the mixture increased by 33% after the addition of EC120 warm mixing agent, and 7% after the addition of Evotherm M1 warm mixing agent, indicating that the enhancement effect of EC120 on the high temperature stability of porous asphalt mixture is better than Evotherm M1.

### 4.3 Low temperature bending test of beam

**Fig 11:**
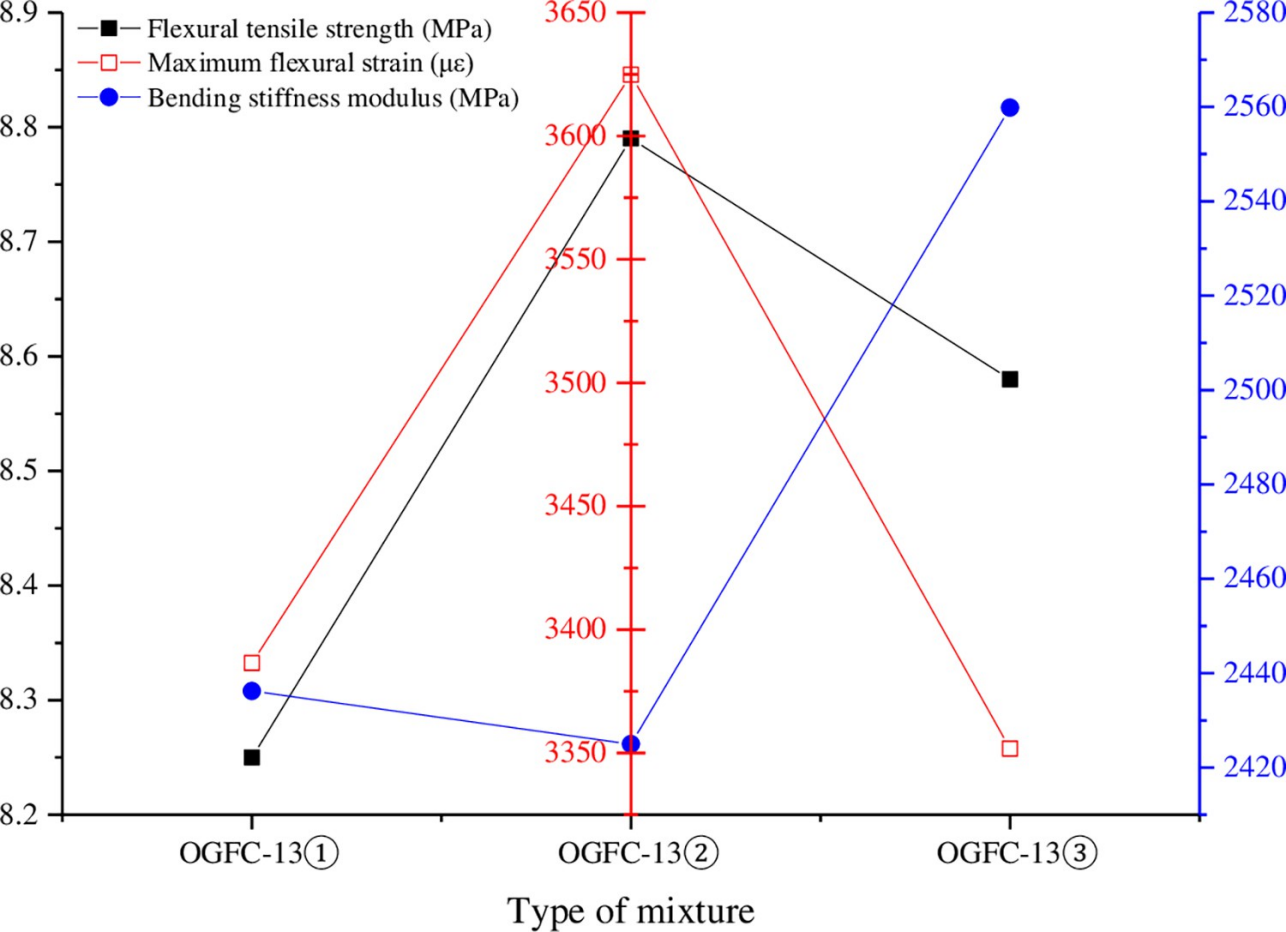
Comparison of low temperature bending test results.

The low temperature bending test results of each mixture are shown in Table 9 and Fig 11.

**Table 9:** Results of low temperature bending test.

Type of mixture	Flexural tensile strength (MPa)	Maximum flexural strain(με)	Bending stiffness modulus (MPa)
OGFC-13①	8.25	3386.42	2436.20
OGFC-13②	8.79	3624.78	2424.97
OGFC-13③	8.58	3351.77	2559.84

As can be seen from Table 9 and Fig 11, the flexural tensile strength is OGFC-13②> OGFC-13③> OGFC-13①, the maximum flexural tensile strain is OGFC-13②> OGFC-13③> OGFC-13①, and the bending stiffness modulus is OGFC-13③> OGFC-13①> OGFC-13②. At low temperature, the greater the maximum flexural tensile strain of the mixture, the better the low temperature cracking resistance, and the maximum flexural tensile strain of each mixture is greater than the requirement of not less than 3000 in cold winter area, indicating that the high viscosity porous asphalt mixture has good low temperature performance. The maximum flexural strain of porous asphalt mixture increased by about 7% after Evotherm M1 warm mixing agent was added, indicating that Evotherm M1 warm mixing agent can improve the low temperature performance of the mixture. The maximum flexural strain of porous asphalt mixture is reduced by about 1% after EC120 warm mixing agent is added, which indicates that EC120 warm mixing agent has a certain weakening effect on the low temperature performance of the mixture.

### 4.4 Water stability test

**Fig 12:**
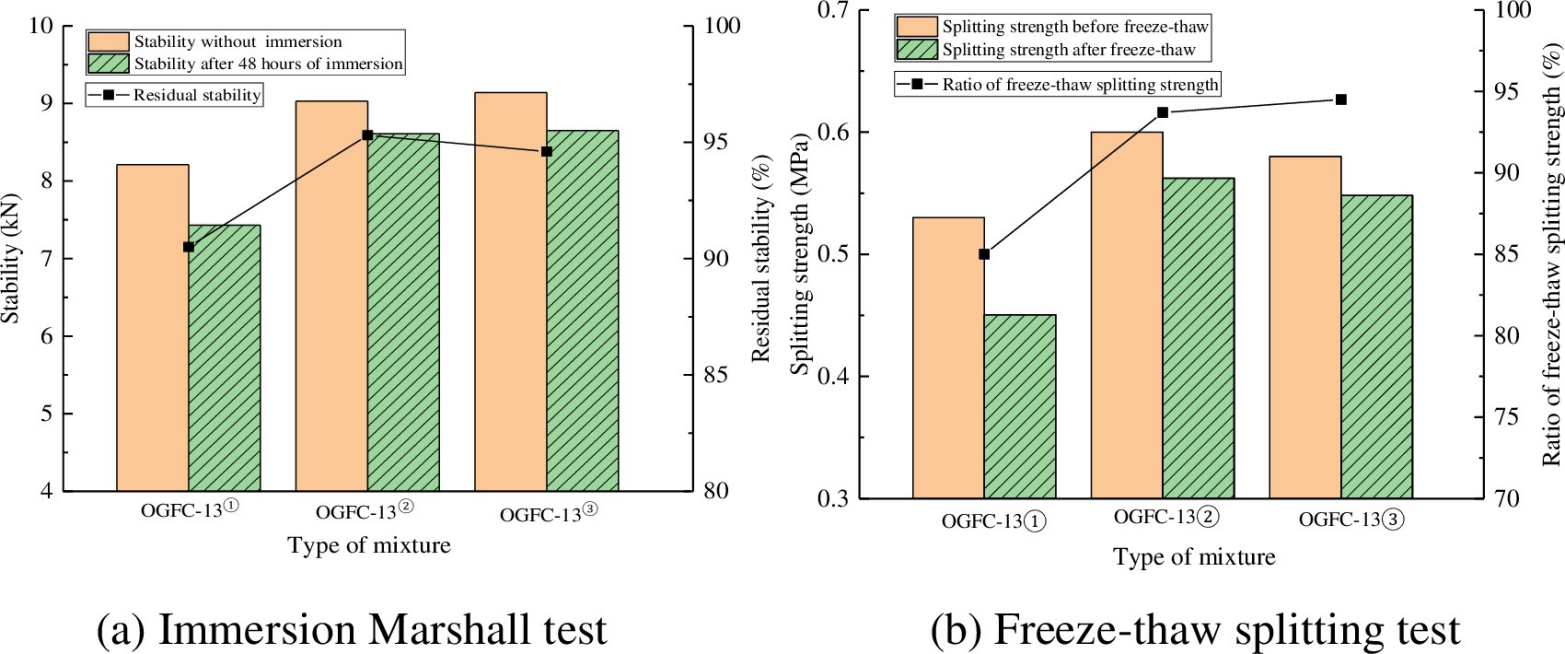
Comparison of water stability test results.

The water stability test results of each mixture are shown in Table 10 and Fig 12.

**Table 10:** Test results of water stability.

Type of mixture	Stability without immersion (kN)	Stability after 48 hours of immersion (kN)	Residual stability (%)	Splitting strength before freeze-thaw (MPa)	Splitting strength after freeze-thaw (MPa)	Ratio of freeze-thaw splitting strength(%)
OGFC-13①	8.21	7.43	90.5	0.53	0.45	85.0
OGFC-13②	9.03	8.61	95.3	0.60	0.56	93.7
OGFC-13③	9.14	8.65	94.6	0.58	0.54	92.5

As can be seen from Table 10 and Fig 12, the stability before and after immersion is OGFC-13③> OGFC-13②> OGFC-13①, and the residual stability is OGFC-13②> OGFC-13③> OGFC-13①. The stability before and after freeze-thaw was OGFC-13②> OGFC-13③> OGFC-13①, and the ratio of freeze-thaw splitting strength was OGFC-13②> OGFC-13③> OGFC-13①. The stability, residual stability, splitting strength and freeze-thaw splitting strength ratio of porous asphalt mixture without warm mixing agent are the smallest, which indicates that the water stability of porous asphalt mixture can be improved by adding warm mixing agent. The ratio of residual stability and freeze-thaw splitting strength is OGFC-13②> OGFC-13③, indicating that Evotherm M1 is better than EC120 for improving the water stability of the mixture.

### 4.5 Uniaxial penetration strength test

**Fig 13:**
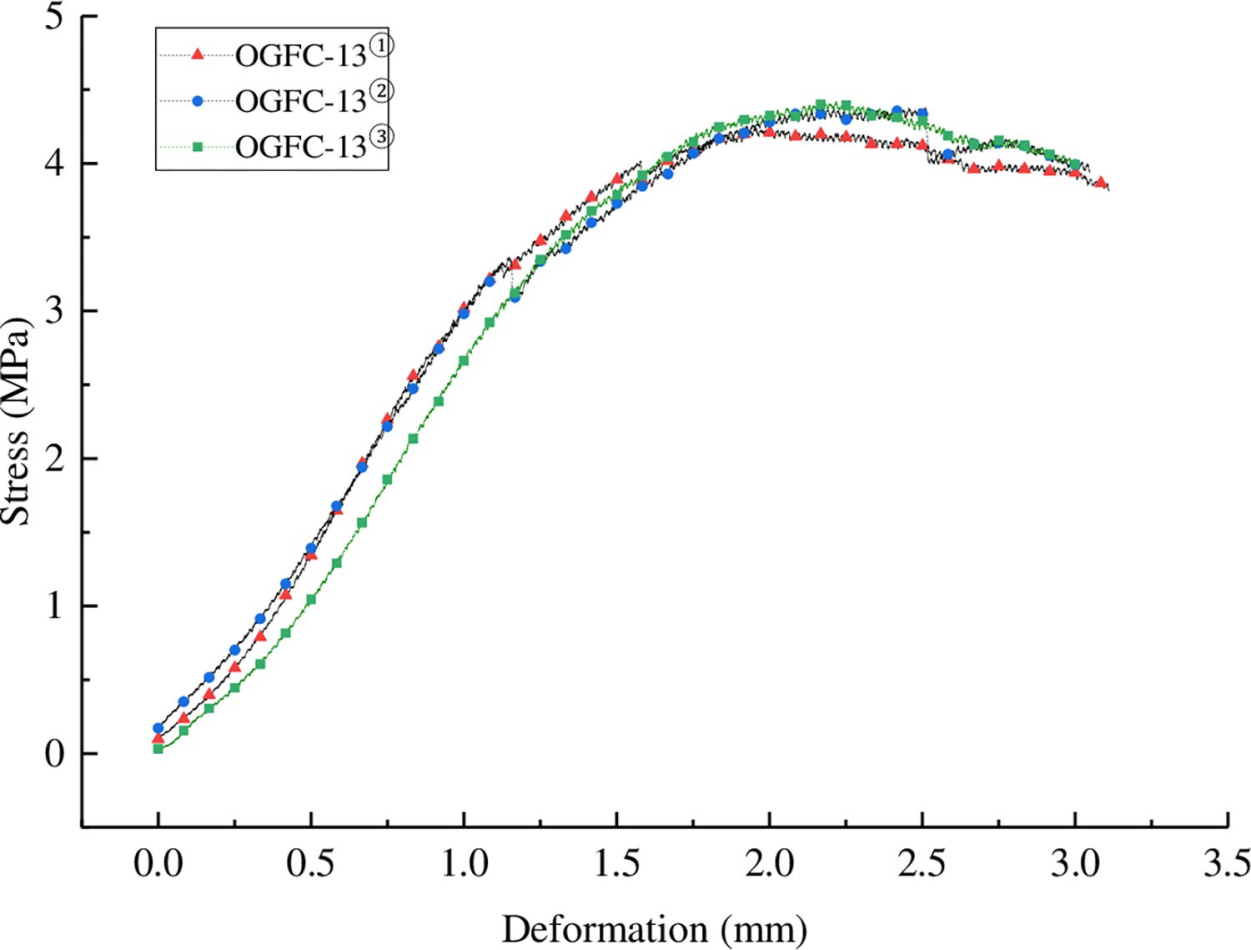
Stress-deformation curve of uniaxial penetration test.

The uniaxial penetration strength test results of each mixture are shown in Fig 13 and Table 11.

**Table 11:** Results of penetration strength test.

Type of mixture	Failure load (N)	Penetration stress (MPa)	Penetration strength (MPa)
OGFC-13①	2703.45	4.238	1.441
OGFC-13②	2791.72	4.376	1.488
OGFC-13③	2818.50	4.418	1.502

It can be seen from Fig 13 and Table 11 that the penetration stress of the mixture increases first and then decreases with the increase of deformation. The peak penetration strength ranked as OGFC-13③> OGFC-13②>OGFC-13①from large to small, which was consistent with the conclusion of rutting test, indicating that the addition of warm mixing agent would increase the shear resistance of the mixture. This is mainly because the addition of warm mixing agent will make the mixture easier to roll and compact during the molding process. The penetration strength of porous asphalt mixture increased by about 3% after the addition of Evotherm M1 warm mixing agent, and increased by about 4% after the addition of EC120 warm mixing agent. The two warm mixing agents have similar enhancement effects on the shear resistance of the mixture.

## 5 Conclusions

Through SGC rotary compaction test, rutting test, low temperature bending test, immersion Marshall test, freeze-thaw splitting test and penetration strength test, the cooling effect of Evotherm M1 and EC120 warm mixing agents on the high viscosity porous asphalt mixture, as well as the influence of high temperature performance, low temperature performance, water stability performance and shear resistance performance have been studied. The main conclusions are as follows:

1. The addition of warm mixing agent can reduce the compaction difficulty coefficient of porous asphalt mixture, and the correlation expression between the compaction difficulty coefficient of asphalt mixture and temperature is obtained by regression. The two warm mixing agents can reduce the mixing and rolling temperature of the mixture to more than 10°C.
2. The addition of warm mixing agent will improve the high temperature stability of porous asphalt mixture, and the effect of EC120 is better than Evotherm M1. Evotherm M1 improves the low temperature performance of porous asphalt mixture, while EC120 has a certain weakening effect. The addition of warm mixing agent can improve the water stability of porous asphalt mixture, and the improvement effect Evotherm M1 is better than EC120.
3. The penetration strength of the warm mixed porous asphalt mixture prepared by rotary compaction is about 3%~4% higher than that without warm mixing agent, and the reinforcement effect of the two warm mix agents is equivalent.
